# Staphylococcal Enterotoxin A Shapes Monocyte Transcription and Macrophage Polarization: Implications for Immune Responses in Infection and Inflammation

**DOI:** 10.1002/eji.70104

**Published:** 2025-12-19

**Authors:** Claudia Arasa, Khaleda Rahman Qazi, David Brodin, Manuel Mata Forsberg, Eva Sverremark‐Ekström

**Affiliations:** ^1^ Department of Molecular Biosciences The Wenner‐Gren Institute, Stockholm University Stockholm Sweden; ^2^ Bioinformatics and Expression Analysis Core Facility Karolinska Institutet Huddinge Sweden

**Keywords:** immune activation, macrophage polarization, superantigens, Staphylococcal enterotoxins

## Abstract

Staphylococcal enterotoxins (SE) crosslink the MHC‐II on antigen‐presenting cells (APC) with the T‐cell receptor, inducing a polyclonal T‐cell response. Although APCs are the initial targets of SE and are critical in shaping subsequent T‐cell activation, the effects of SE on APC function remain poorly understood. This study investigates the immunomodulatory effects of staphylococcal enterotoxin A (SEA) on monocytes and their differentiation into monocyte‐derived dendritic cells (moDC) or macrophages (MDM). Transcriptomic analyses of human monocytes via RNA sequencing revealed SEA‐induced enrichment of gene pathways associated with inflammation, infection, and dermatitis, effects that were amplified in the presence of T cells. Phenotypic and functional characterization showed that SEA‐primed monocytes differentiated into MDM with an altered polarization, deviating from classical M1/M2 pathways. SEA‐primed MDM exhibited downregulation of key markers, including HLA‐DR, CD80, CD86, and PD‐L1. Functional assays demonstrated that SEA‐primed MDM pushed hyperinflammatory T‐cell responses, with significantly enhanced proliferation and IFN‐γ secretion. In contrast, following SEA‐priming, moDC retained robust antigen‐presenting capabilities and displayed enhanced expression of molecules involved in T‐cell interactions. These findings provide mechanistic insights into SEA‐mediated immune modulation, illustrating how SEA reprograms MDM functions and amplifies proinflammatory T‐cell responses. This advances our understanding of superantigen‐driven immune interactions, offering a foundation for developing therapeutic strategies to mitigate superantigen‐mediated immune conditions.

## Graphical abstract

Staphylococcal enterotoxin A (SEA) alters monocyte differentiation and function, while preserving T cell stimulatory capacity. SEA‐primed macrophages downregulate antigen‐presenting markers yet drive heightened T‐cell proliferation and IFN‐γ secretion. These findings reveal mechanisms of SEA‐mediated immune modulation and superantigen‐driven inflammation.

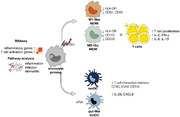

**Abbreviations**
APC: antigen‐presenting cell
CFS: cell‐free supernatants
MDM: monocyte‐derived macrophage
MHC‐II: major histocompatibility complex class II
moDC: monocyte‐derived dendritic cell
SE: Staphylococcal enterotoxin
SEA: Staphylococcal enterotoxin A
TCR: T‐cell receptor
TSS: toxic shock syndrome

## 1 Introduction

*Staphylococcus (S.) aureus*, a gram‐positive bacterium, has a dual role as a commensal and opportunistic pathogen. Approximately 30% of the population is colonized by *S. aureus*–predominantly on the skin, nasopharynx, and in the gut. As a pathogen, it is a public health concern, since it is one of the leading causes of bacterial disease, ranging from superficial skin infections to potentially life‐threatening sepsis. To ensure such a range of pathogenicity, *S. aureus* encodes many different virulence factors, among which are the staphylococcal enterotoxins (SE), which act as superantigens [1, 2].

SE are small proteins that circumvent conventional antigen presentation by cross‐linking the major histocompatibility complex class II (MHC‐II) on antigen presenting cells (APCs) to the T cell receptor (TCR) on T cells outside of the peptide binding groove, inducing polyclonal T cell activation of up to 20% of the T cell pool and releasing cytokines such as IL‐2, TNF, and IFN‐γ [3, 4]. There are currently 26 identified SE and SE‐like proteins, all structurally similar but genetically diverse. Amongst these, SEA is one of the most well‐characterized. It can interact with multiple TCR Vβ chains and the MHC‐II α and β chains, as well as bind to CD28, which further enhances the subsequent T cell activation [5, 6, 7]. SE not only act as superantigens, but also have a separate role as potent gastrointestinal toxins [8]. Excessive cytokine responses to SE can cause systemic inflammatory conditions such as toxic shock syndrome (TSS) [9, 10]. While the advantage of encoding for such a diverse toxin repertoire is unclear, the associated cytokine storm might drive T cell anergy or death, potentially supporting bacterial immune evasion and *S. aureus* persistence. In addition to these pathogenic mechanisms, SE can also cause sensitization. In fact, *S. aureus* colonization and IgE seropositivity to SE are notably higher in patients with atopic conditions compared with healthy individuals [11, 12, 13]. These elevated IgE levels against SE have also been linked to asthma development and exacerbation [14, 15, 16, 17, 18, 19], although the mechanisms underlying this association remain unclear. Furthermore, we have recently shown that allergic individuals display a lower type 1 response to SE compared with nonallergic individuals, highlighting their differential effects in skewed immune systems [20].

Although robust T cell activation driven by superantigens has been well characterized, much less is known regarding their impact on innate immune cells, particularly APCs like monocytes [21, 22, 23]. These cells are among the first responders to pathogens, and they are pivotal in orchestrating inflammation and bridging the innate and adaptive immune responses. Monocytes are circulating cells that, upon receiving inflammatory signals, are capable of complex transcriptional reprogramming, driving their differentiation into specialized cell types, namely monocyte‐derived dendritic cells (moDC) or macrophages (MDM). moDCs are highly specialized in antigen processing and presentation, shaping the adaptive immune responses by directing T cell differentiation. On the other hand, MDM exhibits remarkable diversity and functional plasticity, acquiring different polarization states depending on environmental cues. Traditionally, MDMs have been divided into M1‐like (classically activated) and M2‐like (alternatively activated). Classical activation includes TLR and IFN‐γ stimulation, whereas alternative activation occurs in response to the cytokines IL‐4 and IL‐13. Upon polarization, classically activated MDMs will be involved in pathogen clearance via cytokine secretion and enhanced phagocytic activity, whereas alternatively activated MDMs are associated with tissue repair, clearing of apoptotic cells, and promoting anti‐inflammatory responses. Although this binary classification provides a useful framework, macrophage polarization is not a fixed state but rather a dynamic spectrum influenced by the local microenvironment, emphasizing the adaptability of this cell type [24].

In the last decade, many studies have highlighted the concept of “innate immune training and tolerance” [25, 26], whereby monocyte exposure to microbial stimuli induces lasting functional changes that will shape their subsequent responses. In this study, we investigate the effects of SEA stimulation on monocytes and its immunomodulatory effects on the differentiation and polarization into moDC and MDM, respectively. Furthermore, we also study whether SEA priming of macrophages will influence subsequent T cell responses. By examining the SEA‐priming effect on differentiated myeloid cells and beyond, we aim to elucidate the broader immunomodulatory effects of superantigens.

## 2 Results

### 2.1 SEA Induces a Distinct Transcriptional Shift in HLA‐DR+ Cells, Relevant for Infection, Inflammation, and Dermatitis

**FIGURE 1:**
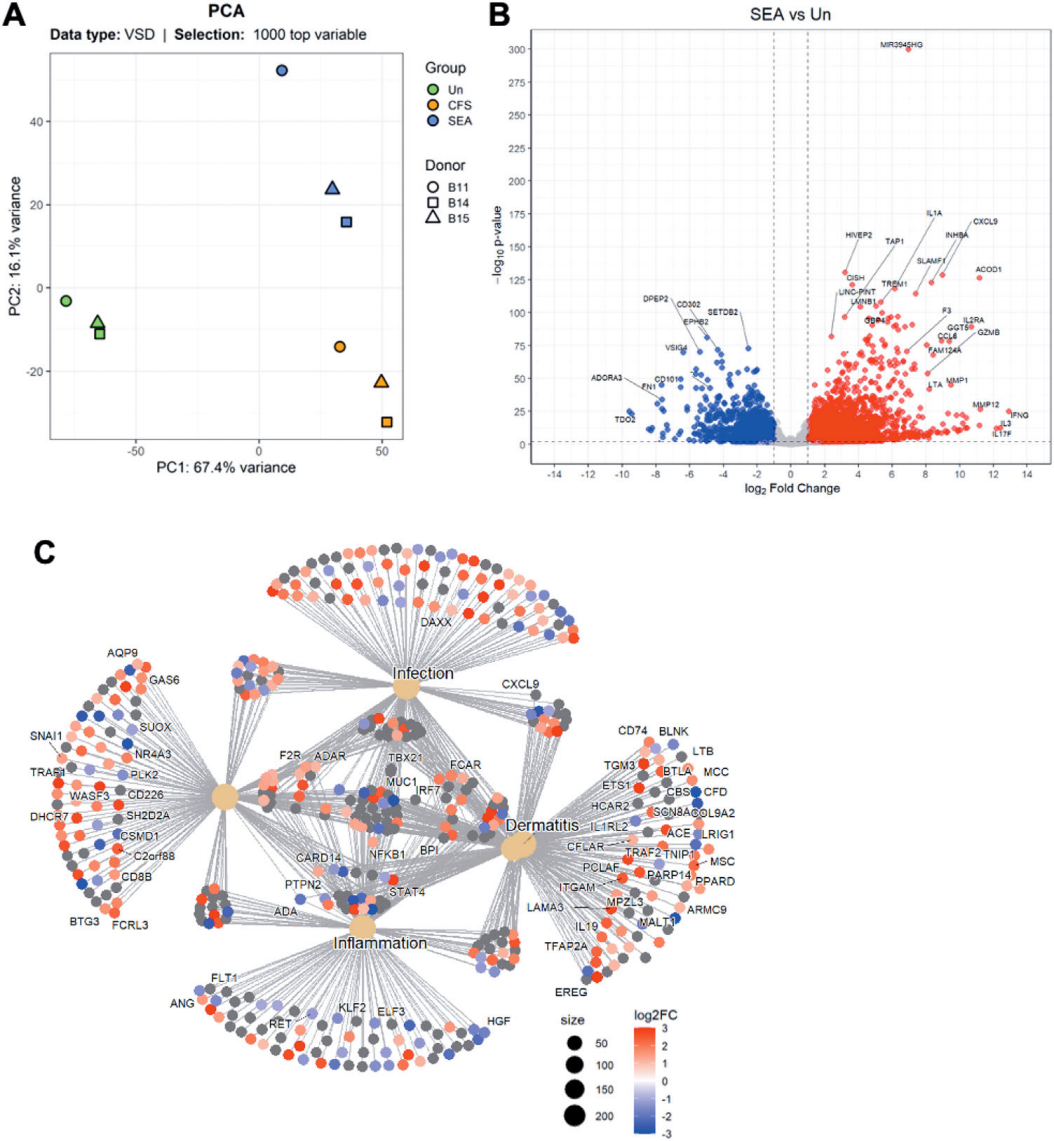
SEA‐induced transcriptional changes in sorted HLA‐DR+ cells from SEA‐stimulated PBMCs. (A) Principal component analysis (PCA) comparing the transcriptional program of HLA‐DR+ cells between unstimulated (green), *S. aureus* CFS‐ or SEA‐stimulated (orange and blue, respectively) upon RNA sequencing. The normalized gene counts of the 1000 most significant genes were used to generate the plot, and the percentage of variance explained by the principal components 1 and 2 (PC1 and 2) is shown on the axes. Symbol shape indicates different donors. (B) Volcano plot comparing the gene expression between unstimulated HLA‐DR+ cells and SEA‐treated HLA‐DR+ cells using the DESeq2 package. (C) DisGeNET enrichment analysis performed with genes with a | log2FC>1 | and p adjusted <0.01 when comparing unstimulated with SEA‐stimulated cells. The 100 most significant genes upon SEA are grouped in their most relevant pathways. Data were collected from three different donors in one experiment.

We aimed to investigate the transcriptional changes induced by SEA in HLA‐DR+ cells during their interaction with T cells. To differentiate the effects attributable to SEA from those induced by other factors, we also used *S. aureus* cell‐free supernatants (CFS) as a control, which represented the broader repertoire of secreted factors, including, but not limited to, SEA. Thus, we stimulated PBMCs with SEA or *S. aureus* CFS for 24 h, then sorted stimulated cells expressing HLA‐DR and negative for other lineage markers (CD3, CD19, CD56, see Figure S1 for a full gating strategy) from the cell mix. We then isolated RNA from the purified HLA‐DR+ cells and sequenced their transcriptional profile. To identify different patterns in the sorted HLA‐DR+ cells, we performed a Principal Component Analysis (PCA) with the 1000 top variable genes (Figure 1A) and observed a remarkable difference between unstimulated and stimulated cells, with one component accounting for 67.4% of the variance. Furthermore, there were also pronounced transcriptional differences between stimulation with isolated toxin and stimulation with the *S. aureus* CFS in the second component, accounting for 16.1% of the variance (Figure 1A). Depicting the most transcriptionally variable genes in a volcano plot (Figure 1B), we observed a complex gene‐specific regulatory response: genes involved in the negative regulation of T cells (such as *VSIG4* and *CD101*), as well as the wound healing gene *FN1*, appeared significantly downregulated. In contrast, other genes also involved in wound healing, like the metallopeptidases *MMO1* and *MMP2*, were significantly upregulated (Figure 1B). Further, we elaborated on some of the genes of interest and created heatmaps showing the magnitude and significance of each gene upon *S. aureus* CFS stimulation or SEA stimulation. Specifically, HLA‐DR+ cells stimulated with SEA upregulated many proinflammatory genes, involved in cytokine signaling such as *CCL7*, *CCL8*, *CXCL1*, *CXCL9*, and *IL15RA*; T cell activation, such as *CD80*, *CD274* (*PDL1*), *IL2RA*, *SLAMF1*, and *TREM1*; and immune regulation, such as *SOCS1*, *GBP5*, *SLAMF1*, *FPR2*, and *ACOD1*. For the most part, the results observed upon *S. aureus* CFS stimulation were similar to those seen upon SEA (Figure S2A). Molecules related to cell adhesion and migration, signal transduction, and structural proteins were also induced by SEA stimulation, amongst which was *OCSTAMP*, osteoclast stimulatory transmembrane protein, and the only gene differently induced by *S. aureus* and SEA (Figure S2B).

To further understand the biological significance of the transcriptional changes induced by SEA treatment in monocytes, we performed a DisGeNET analysis categorizing differentially expressed genes based on their associations with various diseases. The expressed genes were categorized into groups related to infection, dermatitis, and inflammation (Figure 1C). Relevant for infection and inflammation, we observed upregulation of *DAXX*, involved in transcription repression and apoptosis, and *ANG*, involved in angiogenesis. We also detected the downregulation of *RET* (cell growth, differentiation, and survival), *KLF2* (transcription factor for anti‐inflammatory genes), *ELF3* (regulating cytokine and chemokine expression in epithelial cells), and *HGF* (involved in tissue repair during inflammation). Upregulated genes in both infection and inflammation pathways were *F2R*, which links coagulation with inflammation; *ADAR*, crucial in antiviral immune responses; and *FCAR*, involved in IgA responses at mucosal sites.

Related to dermatitis, we found an upregulation of *CD74*, *BTLA*, *TRAF2*, *TNIP1*, and *IL19*. These genes all contribute to immune response regulation as well as TNF and NF‐κB signaling, and their upregulation contributes to the inflammatory responses seen in dermatitis. On the other hand, *IL1R2* and *MALT1*, involved in IL‐1 regulation and NF‐κB signaling, respectively, were downregulated in response to SEA stimulation. *CARD14*, belonging to both inflammation and dermatitis pathways, was also upregulated. So was *STAT4*, which mediates the response to IL‐12, driving the strong Th1 responses characteristic of SEA stimulation.

Last, we also observed changes in the transcription of genes like *GAS6*, crucial in immune regulation and apoptosis inhibition; *NR4A3*, regulator of apoptosis and inflammation; *CD8B*, linked to MHC‐I presentation to cytotoxic T cells; *FCRL3*, involved in B cell activity in autoimmunity; and *BTG3*, involved in cell growth, differentiation, and apoptosis. Furthermore, genes related to cell migration and invasive properties, such as *SNAI1* and *WASF3*, were also upregulated in monocytes upon SEA treatment (Figure 1C).

### 2.2 T Cells Are Required for the SEA‐Induced Transcriptional Response to SEA in Monocytes

**FIGURE 2:**
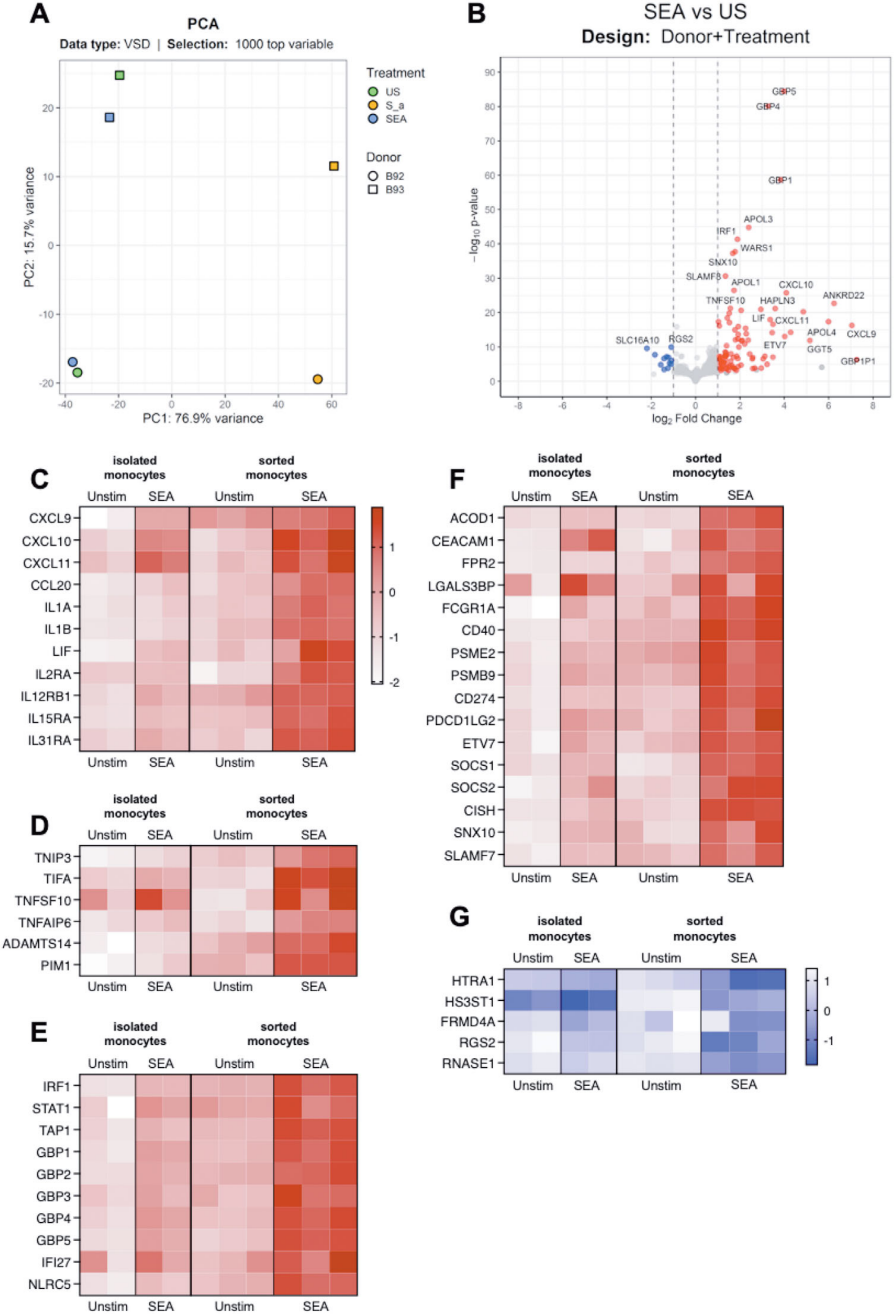
SEA‐ induced transcriptional changes in isolated monocytes in the absence of T cells. (A) PCA of the 1000 most variable genes in unstimulated (green), *S. aureus* CFS‐ (orange), or SEA‐stimulated (blue) isolated monocytes after RNA sequencing. The percentage of variance explained by the principal components 1 and 2 (PC1 and 2) is shown on the axes. Symbol shape indicates different donors. (B) Volcano plot comparing the expression between unstimulated and SEA‐stimulated monocytes. (C–F) Volcano plot showing upregulated genes and (G) downregulated genes in isolated and sorted monocytes. Genes with a | log2FC>1 | and p adjusted <0.01 when comparing unstimulated with SEA‐stimulated cells were selected. Normalized gene counts were first log2‐transformed to reduce data skewness, and then normalized using Z‐scores to place all genes on a unified scale to allow for direct comparison of expression patterns. The resulting visualization illustrates the relative expression of each gene, with red indicating upregulation and blue downregulation. Data were collected from five different donors and two independent experiments: two donors for isolated monocytes and three donors for HLA‐DR+ cells.

We next wanted to investigate whether an encounter with SEA would influence the monocyte transcriptome in the absence of T cells. To do that, we first isolated monocytes using a monocyte enrichment kit and magnetic separation. The isolated monocytes were then exposed to SEA for 24 h. In the absence of T cells, the monocyte transcriptional pattern upon SEA exposure was not as pronounced as when T cells were present, as is summarized in the PCA plot in Figure 2A. Still, some clear differences between unstimulated and SEA‐exposed cells were observed (Figure 2B). The transcription of several genes involved in the interferon and immune response pathways was upregulated, such as the guanylin binding proteins *GBP5*, *GBP4*, and *GBP1*; the chemokines *CXCL9*, *CXCL10*, and *CXCL11*; the transcription factor *IRF1*; and the cytokines *TNFSF10* and *LIF*. We also detected enhanced transcription of the apolipoproteins *APOL1*, *APOL3*, and *APOL4*, which also act as immune regulators in response to infection. On the other hand, expression of *SLC16A10* and *RGS2*, related to amino acid transport and G‐protein signaling, respectively, was downregulated upon SEA stimulation (Figure 2B). To further explore the context of the key genes observed in the volcano plot, we compared their normalized gene counts across three conditions: unstimulated, SEA‐ and *S. aureus* CFS‐stimulated (Figure S2C). Overall, and as expected, we see that the response to *S. aureus* CFS is not solely dependent on SEA. We detected that the significant transcriptional shifts seen in the *CXCL9*, *10*, and *11* chemokines, as well as the guanine‐binding proteins GBP1, 4, and 5, were induced by SEA stimulation and not detected in the CFS‐stimulated cells. The same was true for *TNFSF10*, but not for *IRF1* and *LIF*, which were upregulated upon both stimulations, but to different degrees. Notably, we did not observe an upregulation of any *HLA‐DR* genes upon SEA stimulation in the absence of T cells, whereas these molecules were repressed upon stimulation with the CFS (Figure S2C).

We next compared the transcriptional profiles of isolated monocytes exposed to SEA with those of HLA‐DR+ cells exposed to SEA in the presence of T cells and subsequently sorted. Most of the significantly upregulated genes in isolated monocytes were also upregulated in sorted monocytes. Chemokines, cytokines and their receptors, including *CXCL9*, *CXCL10, CXCL11*, *CCL20*, and cytokine receptors such as *IL12RB1*, *IL15RA*, *IL31RA*, were upregulated (Figure 2C). Genes associated with apoptosis and cell death, such as *TNIP3* and *TIFA* (involved in regulating cell death and inflammation), and *TNFSF10* and *TNFAIP6* (key players in apoptosis), were also upregulated (Figure 2D). Additionally, transcriptional regulators of interferon signaling, *IRF1* and *STAT1*, were significantly upregulated, along with interferon‐induced genes such as *GBP1‐5* and *NLRC5*, linked to inflammasome activation and antimicrobial responses (Figure 2E). Other upregulated genes included those involved in immune regulation (*CEACAM1*, *FPR2*, *LGALS3BP*); co‐stimulatory receptors (*CD40* for activation; *CD274* for repression); and immune suppression (*ETV7*, *SOCS1 SOCS2*, *CISH*) (Figure 2F). Among the significantly downregulated genes were genes involved in amplifying inflammation, inducing tissue damage, and loss of barrier function (*HTRA1*, *HS3ST1*, *FRMD4A*), but also genes that prevent excessive inflammation (*RGS2*, *RNASE1*) (Figure 2G).

### 2.3 MoDC Differentiation Does Not Differ Upon SEA Priming

**FIGURE 3:**
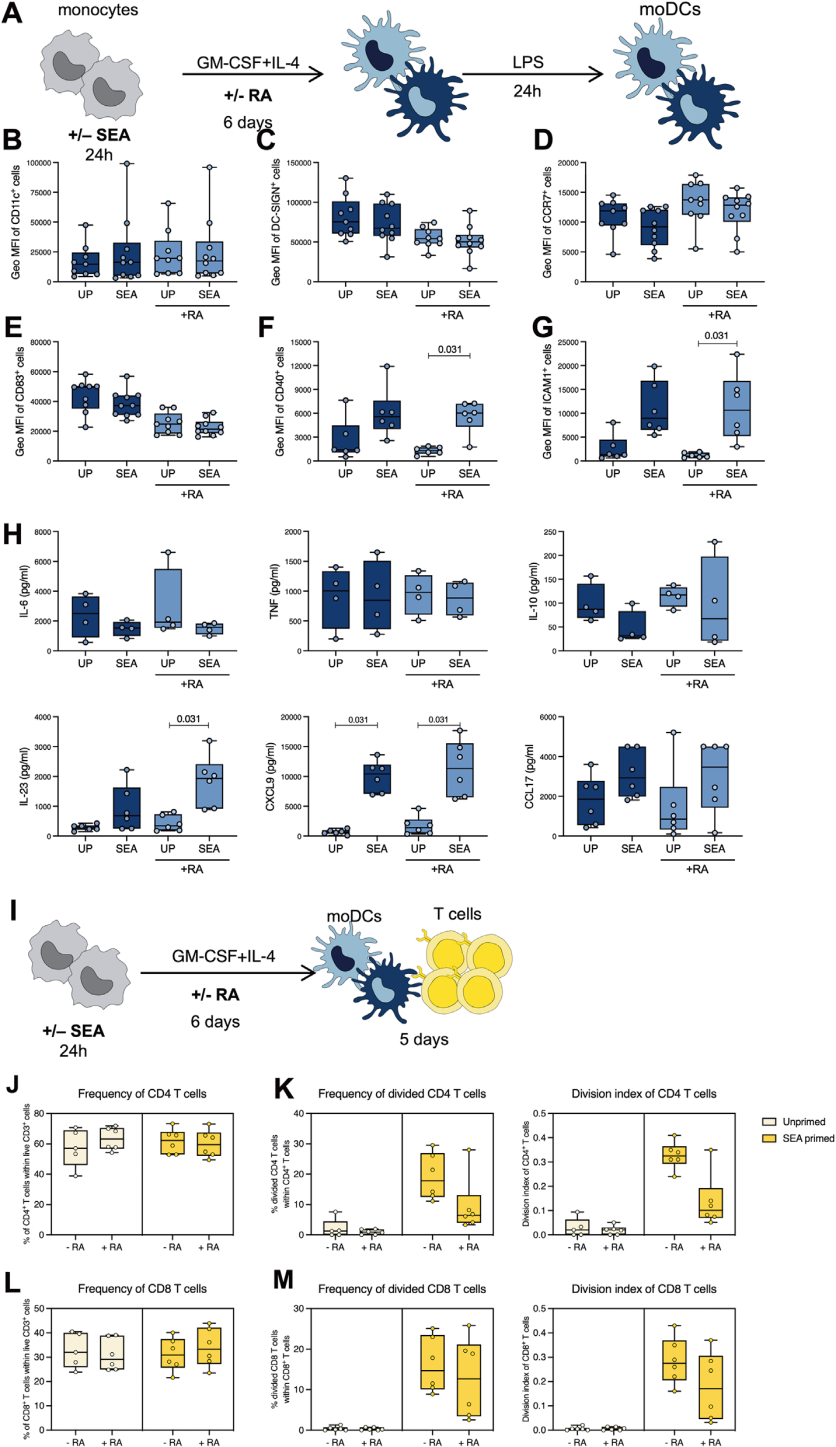
Impact of SEA priming on the differentiation of moDC. (A) Schematic representation of the experimental setup for moDC differentiation. Geometrical mean fluorescence intensity of the surface markers (B) CD11c, (C) DC‐SIGN, (D) CCR7, (E) CD83, (F) CD40, and (G) ICAM1(CD54). (H) Cytokine secretion levels after a 24 h stimulation with 100 ng/ml of LPS. (I) Schematic representation of the experimental setup of moDC co‐culture with autologous T cells. (J) Percentage of CD4+ T cells among total CD3+ cells. (K) Percentage of dividing CD4+ T cells, and their division index. (L) Percentage of CD8+ T cells among total CD3+ cells. (M) Percentage of dividing CD8+ T cells, and their division index. The data are presented as median with interquartile range, with dots representing individual donors. The Wilcoxon matched‐pairs signed rank test was used to determine statistical differences. *p*‐values below 0.05 were considered statistically significant. Data were obtained from six to seven donors and five independent experiments.

As there were subtle but still biologically relevant shifts in monocyte gene expression upon SEA stimulation, we next wanted to investigate whether SEA priming of isolated monocytes would have an effect on subsequent cell differentiation. We preconditioned isolated monocytes for 24 h with SEA, followed by the differentiation into moDC using GM‐CSF and IL‐4, with or without retinoic acid (RA). RA‐differentiated moDCs aimed to mimic gut‐like moDCs. After differentiation, cells were stimulated with LPS for 24 h, and their phenotype and cytokine secretion were analyzed using flow cytometry and ELISA (schematic representation of the experimental setup is shown in Figure 3A).

First, we examined the surface expression of key indicators of differentiation and activation on moDCs derived from SEA‐primed versus unprimed monocytes. No significant differences in expression levels were observed for the markers CD11c (Figure 3B), DC‐SIGN (Figure 3C), chemokine receptor CCR7 (Figure 3D), or activation marker CD83 (Figure 3E). In contrast, there was a marked upregulation of markers important for DC‐T‐cell interactions, such as CD40 (Figure 3F) and ICAM1 (CD54) (Figure 3G). The gut‐like moDC group (Figure 3B–G, in light blue) tended to display different levels of some of the studied markers compared with those differentiated without RA. Still, the overall pattern was similar to that without RA (Figure 3B–G).

To study whether SEA priming affected the functional responses of moDCs, we measured the secreted levels of IL‐6, TNF, IL‐10, IL‐23, CXCL9, and CCL17 (Figure 3H). There were no significant alterations in the secretion of IL‐6, TNF, or IL‐10 in SE‐primed moDCs, although both IL‐6 and IL‐10 appeared to be less induced. In contrast, we observed a SEA‐mediated enhancement of IL‐23 and CXCL9, but also of CCL17. In line with the above findings for surface markers, the addition of RA did not alter the overall pattern of the secreted factors.

Given that the main role of moDCs is to present antigen to T cells, we next investigated the effects of monocyte priming on subsequent co‐culture with T cells. Differentiated moDCs in the presence or absence of RA were thus co‐cultured with autologous T cells labelled with Cell Trace violet. After 5 days of co‐culture, T cell division was addressed using flow cytometry (see Figure 3I for the experimental layout). As shown in Figure 3J, CD4 T cells were found in similar proportions in all co‐culture conditions. However, analyzing the percentage of divided CD4 T cells, as well as their division index, SEA‐primed moDCs induced higher T cell proliferation. Interestingly, co‐culture with gut‐like moDCs led to reduced T cell division compared with co‐culture with regular moDCs (Figure 3K). CD8 T cells were also found in similar proportions regardless of moDC priming (Figure 3L), but the percentage of dividing cells, as well as their division index, was higher when monocytes had been primed with SEA, and similarly in the presence or absence of RA (Figure 3M). Last, T cells in co‐culture with SEA‐primed moDCs secreted IFN‐γ, TNF, IL‐5, and IL‐13. However, IFN‐γ, IL‐5, and IL‐13 were not detected in the supernatants of co‐culture with gut‐like moDCs (Figure S3).

### 2.4 SEA Priming Leads to a Distinct MDM Polarization Pattern That Does Not Translate to Differences in Cytokine Secretion

**FIGURE 4:**
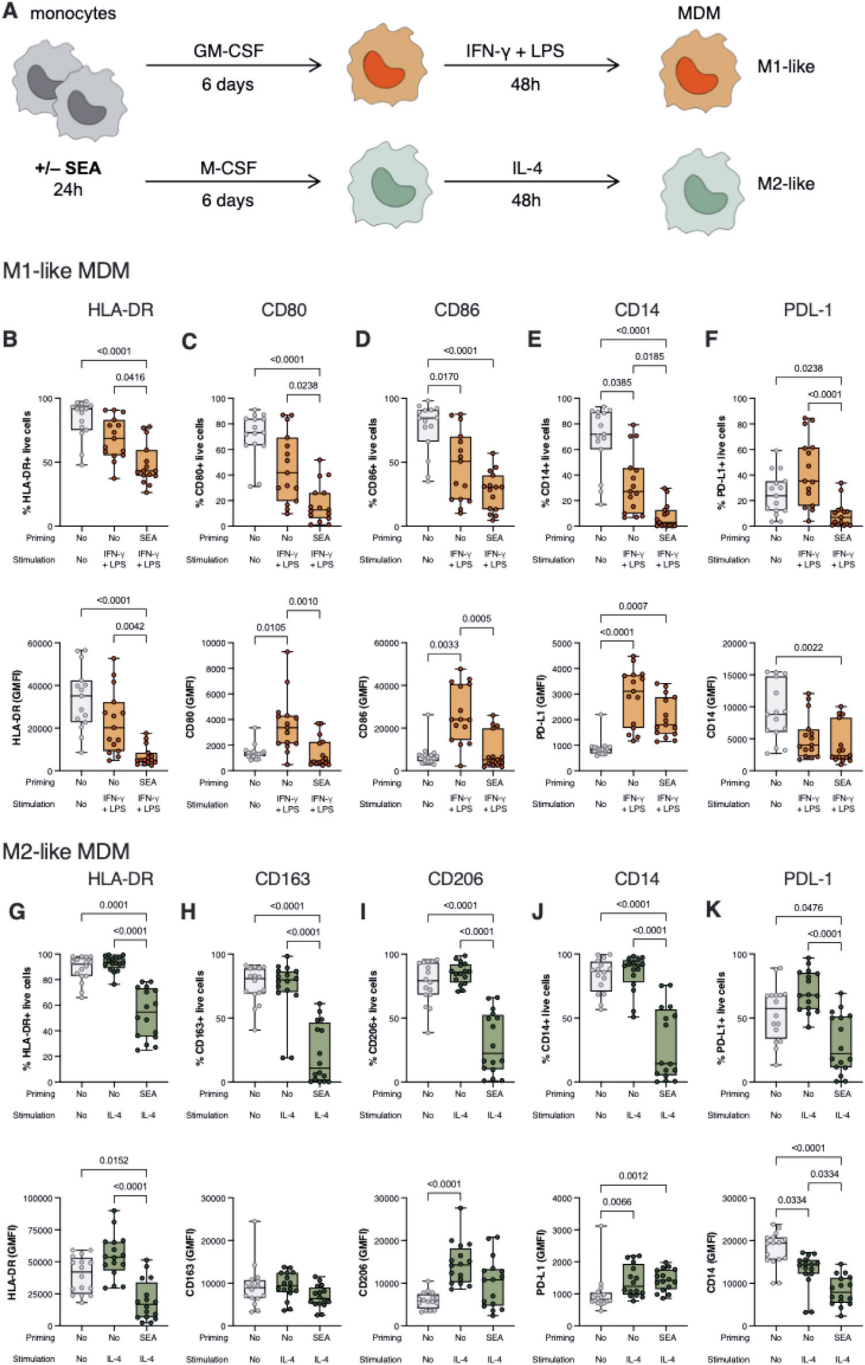
Effect of SEA priming on monocyte‐derived MDM polarization and phenotype. (A) Schematic representation of the experimental setup. Principal component analysis (PCA) comparing the control, unprimed, and SEA‐primed conditions of (B) GM‐CSF‐differentiated, M1‐like MDM or (C) M‐CSF‐differentiated, M2‐like MDM. (D) Percentage of live, GM‐CSF‐differentiated cells expressing HLA‐DR, or (E) CD80, (F) CD86, (G) CD14, (H) PD‐L1. (I) Percentage of live, M‐CSF differentiated cells expressing HLA‐DR, or (J) CD163, (K) CD206, (L) CD14, (M) PD‐L1. The data are presented as median with interquartile range, with dots representing individual donors. The Kruskal–Wallis test was used to determine statistical differences; *p*‐values below 0.05 were considered statistically significant and are written where appropriate. Data were obtained from 15 to 16 donors and 6 independent experiments.

Under inflammatory conditions, monocytes can also differentiate into MDM, so we next wanted to investigate whether MDM derived from SEA‐primed monocytes would be phenotypically and/or functionally different from those derived from unprimed monocytes. To achieve this, we primed isolated monocytes with SEA for 24 h and then differentiated them into MDM in the presence of GM‐CSF or M‐CSF for 6 days. GM‐CSF‐differentiated cells were then classically activated, using IFN‐γ + LPS; whereas M‐CSF‐differentiated cells were alternatively activated, using IL‐4 (summarized in Figure 4A). For simplicity, we named classically‐activated MDM “M1‐like” and alternatively activated MDM “M2‐like”. As a negative control, we also cultured isolated monocytes throughout the length of the experiment, and did not prime them or stimulate them, although they were differentiated in the presence of GM‐ or M‐CSF.

First, we addressed the overall differences in the three groups (control, unprimed, and SEA‐primed). Principal component analysis revealed a clear separation among the three differentiation conditions in M1‐like MDMs (Figure 4B). The control group (unprimed and unstimulated) formed a distinct cluster, well‐separated from the SEA‐primed and unprimed groups along the principal components. Notably, the SEA‐primed samples were also distinct from the unprimed controls along both PC1 and PC2, which explained 43.28% and 25.95% of the total variance, respectively. Similarly, M2‐like MDMs also formed three separated clusters depending on their conditions, with PC1 explaining 57.52% of the differences, and PC2, 13.55% (Figure 4C). Phenotype analysis revealed that all the studied markers were found in nearly all cells in control conditions, except for PD‐L1, which was found in around 20% of the cells differentiated with GM‐CSF and 50% of the cells differentiated with M‐CSF (Figure 4D–M, grey bar). Classical stimulation led to significantly diminished frequency of CD86‐ and CD14‐expressing cells (Figure 4F,G). However, the expression levels of CD80, CD86, and PD‐L1 among the positive population were significantly increased upon stimulation (Figure S4A). We observed that SEA‐priming of monocytes markedly influenced subsequent M1‐like MDM polarization with respect to phenotypic traits. Percentages of expression of HLA‐DR and the activation marker CD80 were reduced compared with those in unprimed cells (Figure 4D,E). Furthermore, the levels of expression of these markers, as well as CD86, were also significantly reduced within the positive population (GMFI) when monocytes had been primed with SEA before their differentiation (Figure S4A). Likewise, the frequencies of macrophages expressing the TLR4 coreceptor CD14 and the immune checkpoint molecule PD‐L1 were lower in the SEA‐primed macrophages (Figure 4G,H), although the expression levels among positive cells were similar to those of the unprimed cells (Figure S4A).

Stimulation of M‐CSF‐differentiated cells with IL‐4 did not significantly alter the percentages of cells expressing any of the addressed markers (Figure 4I–M), although the expression levels of CD206 among positive cells were significantly upregulated (Figure S4B). However, early exposure to SEA altered the phenotype compared with unprimed cells. The percentage of HLA‐DR expression significantly dropped in MDM that had been challenged with SEA before differentiation (Figure 4I). Furthermore, there were fewer cells expressing the M2 markers CD163 and CD206 upon SEA priming (Figure 4J,K), although expression levels did not differ between unprimed and SEA‐primed cells (Figure S4B). Expression of CD14 was less frequent in SEA‐primed macrophages (Figure 4L), whereas the immune checkpoint marker PD‐L1 was found in a lower percentage of MDM when they had been primed with SEA (Figure 4M). Overall, priming with SEA led to a differential expression of traditional phenotyping and activation markers.

**FIGURE 5:**
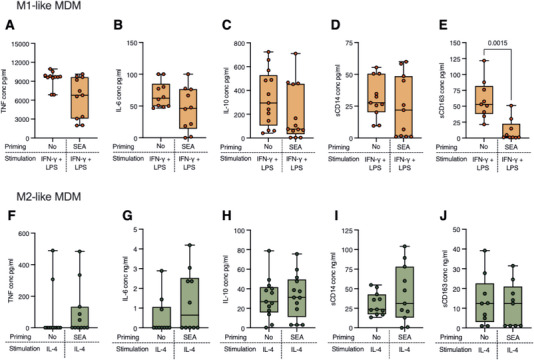
Cytokine and soluble receptor secretion by unprimed and SEA‐primed MDMs under M1 or M2 polarizing conditions. Concentration of (A) TNF, (B) IL‐6, (C) IL‐10, (D) soluble CD14, or (E) soluble CD163 detected in the supernatants of M1‐like polarized macrophages. Concentration of (F) TNF, (G) IL‐6, (H) IL‐10, (I) soluble CD14, or (J) soluble CD163 detected in the supernatants of M2‐like polarized macrophages. The data are presented as median with interquartile range, with dots representing individual donors. Mann–Whitney test was used to determine statistical difference, and *p*‐values below 0.05 were considered significant and are specified where appropriate. Data were obtained from 10 to 13 donors and 6 independent experiments.

To investigate the functional consequences of SEA priming, we also assessed the cytokine secretion profiles of MDM differentiated under the different polarizing conditions by collecting the supernatants after the stimulation shown in Figure 4A. Priming with SEA did not markedly affect the overall cytokine secretion under classical activation conditions. Both SEA‐primed and unprimed MDM secreted comparable levels of the proinflammatory cytokines TNF and IL‐6 (Figure 5A,B), as well as the regulatory cytokine IL‐10 and the soluble co‐receptor sCD14 (Figure 5C,D). However, SEA priming significantly reduced the shedding of the scavenger receptor sCD163 compared with unprimed MDM (Figure 5E). In contrast, when MDM were alternatively activated (upon IL‐4 stimulation), there were no significant differences in the secretion of any studied cytokines or soluble factors, including TNF, IL‐6, IL‐10, sCD14, and sCD163, regardless of their priming status (Figure 5F–J).

### 2.5 MDM Priming Influences T Cell Proliferation and Cytokine Profile

**FIGURE 6:**
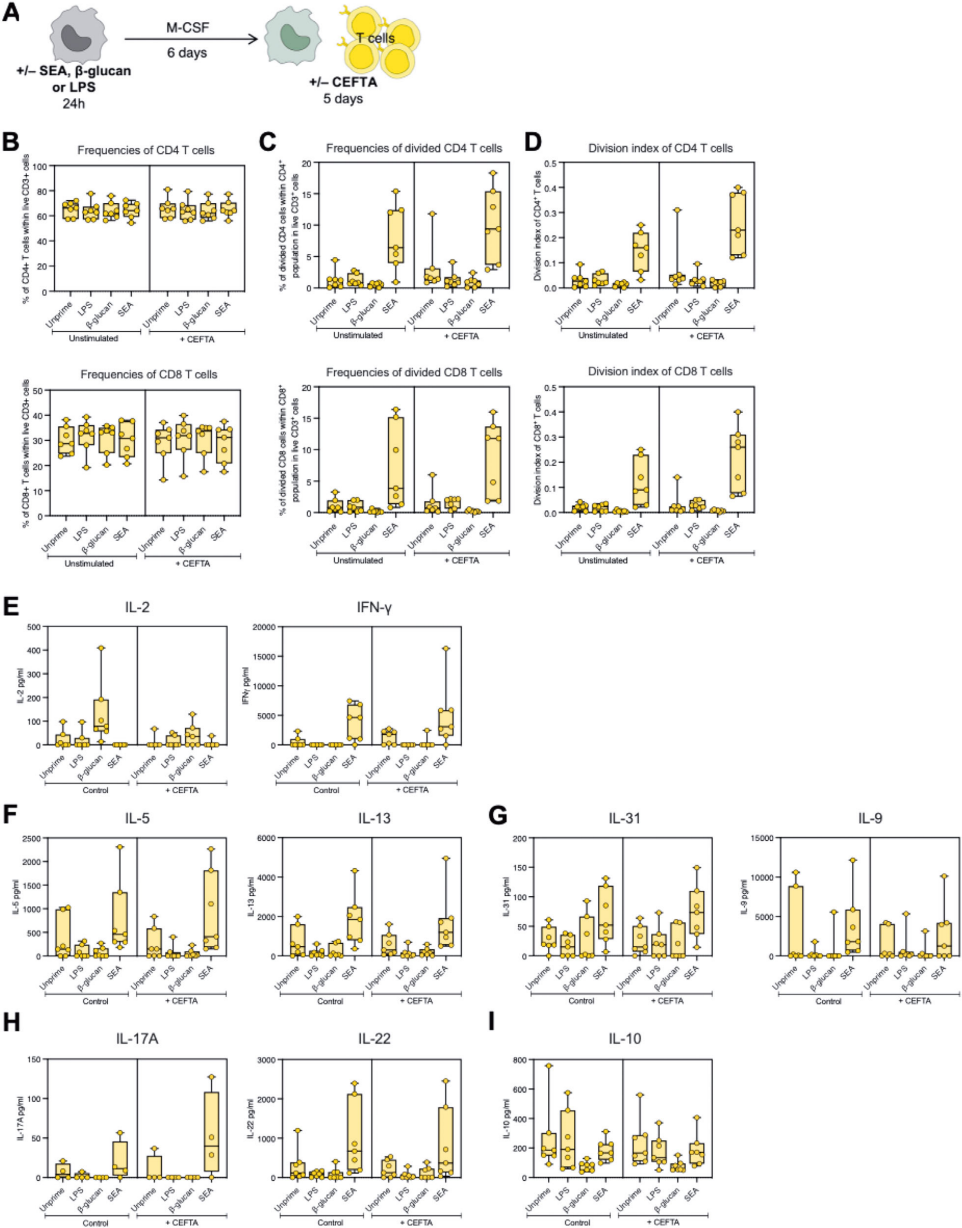
Influence of SEA‐primed, M2‐like macrophages on T cell proliferation and cytokine responses. (A) Schematic representation of the co‐culture setup with differently primed M2‐like macrophages and autologous T cells. (B) Frequency of CD4^+^ (above) and CD8^+^ (below) T cells across the different priming conditions. (C) Frequency of dividing CD4^+^ and CD8^+^ T cells. (D) Division index (average number of cell divisions, including the undivided population) of CD4^+^ and CD8^+^ T cells. Secretion levels of (E) type 1 cytokines IL‐2, IFN‐γ, TNF; (F) type 2 cytokines IL‐5 and IL‐13; (G) IL‐9 and IL‐31, (H) type 3 cytokines IL‐17A and IL‐22; and (I) IL‐10 in the supernatants of the co‐culture. The data are presented as median with interquartile range, with dots representing individual donors. Kruskal–Wallis test was used to determine statistical differences; *p*‐values below 0.05 were considered statistically significant and are written where appropriate. Data were obtained from 7 donors and 3 independent experiments.

MDM, as professional antigen‐presenting cells, are crucial in modulating T cell responses. To investigate the effect of priming with SEA on subsequent T cell responses, we chose to generate macrophages in the presence of M‐CSF (M2‐like MDM), which mimic in vivo homeostatic conditions, and co‐culture with autologous T cells stained with Cell Trace violet. The cells were either kept unstimulated or stimulated in the presence of a peptide pool containing 35 MHC‐II‐restricted peptides from common viruses, designed to stimulate CD4^+^ T cells with a broad array of HLA types (CEFTA) for 5 days. For comparative purposes, monocytes were also primed with β‐glucan or LPS before differentiation, serving as training and tolerance controls, respectively (Figure 6A).

We observed consistent percentages of CD4^+^ and CD8^+^ T cells across all priming and stimulation conditions (Figure 6B). However, the percentage of proliferating T cells was markedly higher in co‐cultures with SEA‐primed macrophages compared with unprimed, β‐glucan‐ or LPS‐primed. This was true across both CD4^+^ and CD8^+^ subsets, regardless of stimulation (Figure 6C). Further analysis of the division index, which represents the average number of divisions that all cells have undergone (including the nondividing cells), confirmed that T cells exposed to SEA‐primed macrophages underwent more divisions than all the other conditions (Figure 6D).

In terms of functionality, IL‐2 was not detected following co‐culture with SEA‐primed MDM and T cells after 5 days of culture (Figure 6E). However, at an earlier timepoint (16 h), SEA‐specific induction of IL‐2 was shown both intracellularly and in the culture supernatants (Figure S5A,B). IFN‐γ and TNF secretion, on the other hand, were prominently induced in T cells co‐cultured with SEA‐primed MDM, regardless of stimulation. On the other hand, β‐glucan‐ or LPS‐primed MDM did not support IFN‐γ secretion from T cells under unstimulated or CEFTA‐stimulated conditions (Figure 6E). The main type 2 cytokines IL‐5 and IL‐13 were selectively produced by T cells interacting with SEA‐primed MDM, although IL‐13 secretion showed high donor variability, with some unprimed MDM, inducing IL‐13 secretion to similar levels (Figure 6F). IL‐9 and IL‐31, often referred to as type 2 cytokines, were also detected in this setting. IL‐31 was produced at low levels, with a tendency of higher secretion when MDM had been primed with SEA; whereas IL‐9 was secreted in high amounts, and the response was similarly higher between unprimed and SEA‐primed macrophages compared with the traditional training or tolerizing conditions (Figure 6G). Additionally, the levels of type 3 cytokines IL‐17A and IL‐22 were elevated in SEA‐primed conditions, both in the control and the stimulated conditions, although CEFTA seemed to increase the levels of IL‐17A secretion compared with the unstimulated (Figure 6H). Lastly, we also measured the secretion of IL‐10, which was detected at low levels and appeared similar in all conditions except for training with β‐glucan, which seemed to suppress this regulatory cytokine (Figure 6I).

## 3 Discussion

In this study, we aimed to elucidate the role of SEA on myeloid cells, a critical yet so far under‐investigated area in understanding superantigen biology. Our results provide new insights into the SEA‐mediated effects on monocyte transcription and activation, but also on the subsequent phenotypic and functional changes in the roles of differentiated APCs, particularly in moDC and macrophages. From our data, it is clear that HLA‐DR+ cells (including circulating monocytes and cDCs) respond most strongly to SEA in the presence of T cells, which, with their strong activation, provide a positive feedback loop that enhances their activation. However, we further demonstrate that isolated monocytes also change their transcriptional profile in response to SEA. Additionally, priming of monocytes leads to transcriptional changes that do not translate into the surface marker expression or function of moDC but do have an impact on the macrophage phenotype and their ability to activate T cells. This differential susceptibility underscores the selective nature of SEA's modulatory effects within the immune system.

HLA‐DR+ cells stimulated within the PBMC population shift their transcriptional profile upon SEA stimulation, endorsing T cell activation and promoting inflammation. This is achieved not only by the upregulation of proinflammatory genes such as cytokines, chemokines, and costimulatory molecules, but also by the downregulation of regulatory genes, collectively promoting T cell activation and inflammation. Furthermore, this transcriptional profile also displayed alterations in wound‐healing gene expression, with fibronectin being downregulated, but matrix metallopeptidases, involved in angiogenesis, being upregulated. These changes suggest a complex role for SEA in modifying myeloid cell function beyond classical inflammation. Gene pathway analysis of SEA‐stimulated HLA‐DR+ cells revealed enrichment in pathways associated with infection, inflammation, and dermatitis, which supports the active role enterotoxins play in *S. aureus* pathogenesis [27]. Epidemiological associations between staphylococcal enterotoxin seropositivity and the development of diseases such as nonallergic asthma and atopic dermatitis have long been recognized [6, 15, 19, 28]. However, to the best of our knowledge, our study is among the first ones to provide a mechanistic insight, showing that SEA primes myeloid cells to foster a proinflammatory microenvironment upon subsequent differentiation.

The transcriptional changes observed in monocytes exposed to SEA, both in the presence and absence of T cells, reflect the complex immune responses elicited by superantigens. The upregulation of chemokines and cytokine receptors highlights SEA's ability to recruit and activate immune cells. Furthermore, activation of interferon regulators such as *IRF1* and *STAT*, as well as interferon‐related genes, emphasizes the strong activation of the immune system that has been reported on T cells [29, 30, 31, 32]. In addition to promoting immune cell activation, SEA also appeared to play a role in balancing immune activation and suppression via the upregulation of cell death and apoptosis (*TNIP2*, *TIFA*) [33, 34], immune regulation and inhibition of costimulatory receptors (such as *CEACAM1* and *CD274* (*PDL‐1*), respectively) [35, 36, 37] and immune suppression (*ETV7*, *SOCS1* and *2*) [38, 39]. Simultaneously, the downregulation of genes that amplify inflammation (*HTRA1*, *HS3ST1*, *FRMD4A*) [40, 41] reflects a potential mechanism to prevent excessive inflammation. Such regulatory dynamics may be essential in preventing tissue damage and maintaining immune homeostasis during SEA exposure.

Notably, the transcriptional profile of SEA‐stimulated monocytes was much stronger and broader when T cells were present during stimulation. This enhanced response likely reflects the critical role of monocyte‐T cell crosstalk, whereby T cell‐derived cytokines and interactions intensify both pro‐ and anti‐inflammatory pathways in monocytes [42]. These findings emphasize that T cell interactions are pivotal in shaping the immune response to SEA, and highlight the interplay between innate and adaptive immunity.

While the interplay between monocytes and T cells clearly amplifies the immune response, our study focused on the priming of isolated monocytes as a model to elucidate the effects of early monocyte‐SEA interaction before their differentiation to downstream myeloid cell types. Despite monocytes being capable of differentiating into both moDC and MDM, the priming effects of SEA on the resulting cell populations were different, and likely rooted in their functionality and distinct differentiation pathways [43, 44].

moDCs are highly specialized in antigen presentation, particularly to naïve T cells, and typically exhibit higher levels of HLA‐DR compared with MDM, which reinforces their capacity to initiate a robust adaptive immune response. Their expression of markers, such as DC‐SIGN, CD40, and ICAM1 (CD54), further enhances their T cell stimulatory capacity, whereas the maturation marker CD83 serves to stabilize their immune regulatory function, ultimately promoting efficient T cell activation [45]. In our experimental conditions, SEA priming of moDCs did not hamper the expression of canonical moDC surface markers but enhanced markers involved in interactions with T‐cells, such as CD40 and ICAM1 (CD54). SEA‐priming of moDCs also enhanced the production of T‐cell‐attracting factors such as CXCL9, CCL17, and IL‐23, which enhances a proinflammatory Th17 response. Collectively, this shows that SEA‐exposed monocytes will differentiate into moDCs that retain their capacity to deliver costimulatory signals to T cells and have the potential to drive proinflammatory T cell responses. We also observed that SEA priming of moDCs markedly enhanced T‐cell proliferation, but that RA decreased this effect, particularly in CD4 T cells. This effect is likely attributable to residual SEA remaining bound to the moDCs, given the toxin's high stability and resistance to degradation [46]. Likewise, IFN‐γ was produced at higher levels in co‐cultures with SEA‐primed moDCs, but was not detected in the co‐cultures with gut‐like moDCs. This is likely due to the mimicking of the gut mucosal environment, which must balance immune defence with tolerance to food antigens and commensal microbes [26].

Relative DCs, MDM perform a broader range of functions, including antigen presentation, pathogen elimination, tissue repair, and maintaining tissue homeostasis [47, 48]. This functional versatility might render them more responsive to environmental cues such as SEA, which we have shown altered their surface marker expression and modulated their interactions with other immune cells, namely, total T cells. We showed that SEA priming influenced the MDM phenotypic markers, partially deviating them from the classical M1 or M2 polarization. Generally, for both M1‐ and M2‐like macrophages, SEA priming led to the downregulation of HLA‐DR, along with the expression of co‐stimulatory markers CD80 and CD86, potentially limiting T cell activation. Despite these shifts, the cytokine profile of SEA‐primed MDM upon a secondary stimulus—including TNF, IL‐6, and IL‐10—remained largely comparable to that of unprimed MDM, suggesting that SEA priming minimally affects the MDM's cytokine secretion. Overall, these findings underline the oversimplification when reducing MDMs to two phenotypes and highlight their plasticity and ability to react to environmental cues. When in a co‐culture setting with autologous T cells, SEA‐primed M2‐like MDM elicited heightened T cell proliferation and IFN‐γ secretion, even with no stimulation. Interestingly, while IL‐2 was not detected in the supernatants of SEA‐primed co‐cultures at the end of the culture after 5 days, we did indeed detect a clear early IL‐2 response specifically in the SEA‐primed co‐cultures after 16 h. We speculate this is due to this cytokine's autocrine properties and the fact that T cells rapidly produce and consume IL‐2 during proliferation, which was only observed in SEA‐primed co‐cultures. These cytokine differences are in contrast with the reduced HLA‐DR and costimulatory molecule expression induced by SEA priming, but might be attributed to the observed reduction in PD‐L1 and CD163, as well as SEA still being bound to the resulting MDMs and binding the TCR on T cells. Furthermore, PD‐L1 is a well‐known immune checkpoint [36, 49], whereas CD163 is linked to reduced lymphocyte activation and proliferation [50]. Their downregulation may hinder the T cell regulation and seems to promote a hyperinflammatory T cell response, exceeding the response elicited by unprimed MDM or MDM primed with the traditional training or tolerance stimuli.

The altered phenotype of SEA‐primed MDM may be particularly relevant in tissues prone to *S. aureus* colonization, such as the skin. SEA priming could be driving a heightened immune state, whereby MDMs seem to have lost typical activating and regulatory functions. This is suggested by their altered expressions of HLA‐DR and co‐stimulatory signals and the downregulation of CD163 and PD‐L1, particularly in the M2‐like phenotype. This would, in turn, hinder their ability to mitigate excessive T cell activation and inflammation in favor of sustained local inflammation, as evidenced by the increased IFN‐γ production. In the context of skin conditions such as atopic dermatitis, where T cells are continuously activated in response to skin barrier dysfunction and bacterial colonization, the presence of SEA‐primed MDM could exacerbate and potentially worsen disease severity.

In conclusion, our findings enhance the understanding of SEA's mechanism of action on myeloid cells, emphasizing not only the importance of its engagement with adaptive immune cells but also its role as a priming agent. By elucidating SEA's selective effects on monocyte differentiation and function, this study provides a foundation for future research aimed at unraveling the broader implications of enterotoxins in immune modulation. This knowledge could inform therapeutic strategies to manage superantigen‐driven immune responses, such as those seen in TSS, with the potential to improve clinical outcomes.

## 4 Data Limitations and Perspectives

Our study provides valuable insights into the immune response triggered by SEA in monocytes and their interaction with T cells; however, some limitations warrant consideration. We primed isolated monocytes to differentiate into moDCs or MDMs. Notably, monocytes within the PBMC context exhibited a more robust response to SEA, suggesting that the resulting differentiated cells from such a setting would likely display a more pronounced and altered phenotype. While investigating SEA's effects on APCs in a broader immune context would be highly informative, the use of isolated monocytes in our setup allowed us to circumvent the overwhelming T‐cell‐mediated inflammatory response. This approach enabled us to focus on the monocyte response to SEA without the confounding effects of a strong cytokine storm.

This strategy is particularly relevant given that *S. aureus* is a common commensal organism. Although SEA and other staphylococcal superantigens are capable of inducing severe conditions such as TSS, many individuals encounter these toxins asymptomatically. Understanding how SEA modulates immune cells in the absence of overt inflammation provides valuable insights into how such toxins may subtly influence immune function in otherwise healthy individuals.

## 5 Materials and Methods

### 5.1 Monocyte Isolation and Stimulation

Peripheral blood mononuclear cells (PBMCs) were isolated from buffy coats of healthy donors by density gradient centrifugation using Ficoll‐Paque PLUS (Cytiva). Monocytes were isolated from fresh PBMCs in two different ways: sorting or CD14^+^ cell enrichment.

Monocytes were sorted from freshly isolated PBMCs that had been stimulated with 20 ng/mL of SEA for 24 h. After incubation, cells were stained with fluorescent antibodies (see sorting panel on Table 1). Sorting was performed using the FACSMelody instrument (BD Biosciences).

**TABLE 1:** Overview of the antibodies used in the experimental setup: Summary of markers, fluorochromes, clones, suppliers, and experimental panels in which each antibody was used.

Marker	Fluorochrome	Clone	Company	Catalog no	Panel
CCR7	BV510	G043H7	BioLegend	353232	moDC
CD3	BV510	UCHT1	BioLegend	300448	Sorting
CD3	PE‐Cy7	SK7	BioLegend	344816	Co‐culture
CD4	PerCP‐Cy5.5	OKT4	BioLegend	317428	Co‐culture
CD8	APC	SK1	BioLegend	344722	Co‐culture
CD11c	APC	3.9	BioLegend	301613	moDC
ICAM‐1	APC	HA58	BD Biosciences	561899	moDC
CD40	PE‐Cy7	5C3	BD Biosciences	561215	moDC
CD14	FITC	M5E2	BD Biosciences	555397	MDM, moDC
CD19	BV421	HIB19	BioLegend	302234	Sorting
CD56	APC	B159	BD Biosciences	555518	Sorting
CD80	PE‐Cy7	2D10	BioLegend	305218	MDM
CD83	PE‐Cy7	HB15e	BioLegend	305325	moDC
CD86	PE	2331 (FUN‐1)	BD Biosciences	560957	MDM, moDC
CD163	APC	GHI/61	BioLegend	333610	MDM
CD206	PerCP‐Cy5.5	15‐2	BioLegend	321122	MDM
DC‐SIGN	BV421	9E9A8	BioLegend	330118	moDC
HLA‐DR	APC‐Cy7	L243	BioLegend	307618	MDM
HLA‐DR	PerCP	L243	BioLegend	307628	moDC, sorting
PDL‐1	BV421	MIH1	BD Biosciences	563738	MDM
IL‐2	BV421	5344.111	BD Biosciences	562914	Co‐culture
Aqua live/dead			Invitrogen	L34966	MDM
Fixable viability Dye 780			Invitrogen	65‐0865‐14	Co‐culture, moDC, sorting
Cell trace violet			Invitrogen	C34557	Co‐culture

Isolated monocytes were obtained by enrichment of freshly isolated PBMCs using the EasySep Human Monocyte Enrichment Kit (STEMCELL Technologies) following the manufacturer's recommended protocol. Briefly, 5 × 10⁷ PBMCs/mL were resuspended in PBS with 2% FCS and 1 mM EDTA (Invitrogen, NY, USA), incubated with CD14 enrichment antibody cocktail, and mixed with magnetic particles. Monocytes were isolated by negative selection with an EasySep magnet and resuspended in RPMI‐1640 medium supplemented with 10% heat‐inactivated fetal bovine serum (FBS), 100 U/mL penicillin, 100 µg/ml streptomycin, and 2 mM L‐glutamine (all from Cytiva). Then, cells were seeded at a 10^6^ monocytes/mL and left untreated or primed with 20 ng/mL of SEA (Toxin Technologies), 2.5% *S. aureus* CFS (in‐house produced); 100 µg/mL whole β‐glucan particles from *Saccharomyces cerevisiae* (InvivoGen), or 100 ng/mL LPS from *Escherichia coli* 055:B5 (Sigma‐Aldrich) for 24 h at 37°C, 5% CO_2_, depending on the experimental setup.

### 5.2 Generation of Bacterial Cell‐Free Supernatants

*S. aureus* 161:2 was grown in Brain Heart Infusion broth (Merck, Darmstadt, Germany) for 72 h at 37°C as a static culture. The bacteria were then centrifuged at 3400*g*, and the supernatants were separated from the pellet. After that, the supernatants were sterile‐filtered (0.2 µm) and stored at −20°C until used.

### 5.3 RNA Sequencing of Monocytes

Total RNA from isolated monocytes was extracted using the mirVana Isolation kit (Ambion by Life Technologies), as per the manufacturer's instructions. RNA quality was assessed using the Agilent TapeStation (Agilent Technologies). Library preparation followed the Illumina Stranded mRNA Prep Ligation protocol (Illumina), involving mRNA isolation, cDNA synthesis, anchor ligation, library amplification, and indexing. Library yield and quality were checked with Qubit (Thermo Fisher Scientific) and the Agilent TapeStation. Libraries were then normalized, pooled, and sequenced on the Illumina NextSeq 550 platform using v2 75‐cycle, high‐output, single‐end mode.

### 5.4 moDC Differentiation and Stimulation

Enriched monocytes were washed after priming, and subsequently differentiated into moDC over six days in the presence or absence of 1.15 µg/mL of all‐trans retinoic acid (RA, Sigma‐Aldrich), to mimic a gut‐like environment. To differentiate them, the cells were cultured in complete RPMI‐1640 medium with 50 µM β‐mercaptoethanol and 2% sodium pyruvate (both from Gibco) containing 50 ng/mL of granulocyte‐macrophage colony‐stimulating factor (GM‐CSF, PeproTech, Rocky Hill, NJ) and 35 ng/mL IL‐4 (PeproTech). Fresh medium containing GM‐CSF, IL‐4, with or without RA was added on day 3. After differentiation, the resulting immature moDC were challenged with 100 ng/mL of lipopolysaccharide (LPS, Sigma‐Aldrich) for 24 h at 37°C, 5% CO_2_, to induce maturation, activation, and cytokine secretion. These were analyzed by harvesting the cells and staining them for cell surface markers (see moDC panel on Table 1), and the expression levels were measured using flow cytometry using FACSVerse (BD Biosciences). Gating was performed, adhering to the guidelines. Furthermore, supernatants were collected and cytokine secretion was measured using ELISA (see Table 2 for reagents).

**TABLE 2:** ELISA kits used in the experimental setup: Summary of analytes and suppliers used.

Analytes	Company	Catalog No
IFNγ	MABTECH	3420‐1A‐6
IL‐2	MABTECH	3445‐1A‐6
IL‐5	MABTECH	3490‐1A‐6
IL‐6	MABTECH	3460‐1A‐6
IL‐9	R&D	DY209‐05
IL‐10	MABTECH	3430‐1A‐6
IL‐13	MABTECH	3471‐1H‐6
IL‐17A	MABTECH	3520‐1A‐6
IL‐22	MABTECH	3475‐1A‐6
IL‐31	MABTECH	3530‐1A‐6
TNF	MABTECH	3512‐1A‐6
IL‐23	MABTECH	3457‐1A‐6
CXCL9	R&D	DY392‐05
CCL17	R&D	DY364‐05

### 5.5 Macrophage Differentiation and Polarization

After priming enriched monocytes for 24 h, the cells were washed with warm RPMI and differentiated into MDM under two different growth conditions with the growth factors. For the generation of the pro‐inflammatory, M1‐like phenotype, primed monocytes were cultured in complete RPMI‐1640 medium containing 50 ng/mL GM‐CSF (PeproTech). To achieve the anti‐inflammatory M2‐like phenotype, primed monocytes were cultured in complete RPMI‐1640 containing 50 ng/mL macrophage colony‐stimulating factor (M‐CSF, PeproTech). Cells were then incubated for six days for differentiation, with the addition of fresh medium containing their respective growth factors on day three. Following differentiation, M1 conditioned macrophages were polarized with 20 ng/mL IFN‐γ (PeproTech) together with 100 ng/mL LPS (Sigma‐Aldrich), whereas M2 conditioned macrophages were polarized in the presence of 20 ng/mL IL‐4 (PeproTech) for 48 h to induce pro‐ and anti‐inflammatory‐like phenotypes, respectively. After polarization, the cells were harvested and stained for downstream analysis (see MDM panel on Table 1), including surface marker expression using flow cytometry using FACSVerse (BD Biosciences). Gating was performed adhering to the guidelines, and cells showed good viability (Figure S6A,B). Cell culture supernatants were analyzed for the cytokines using ELISA (see Table 2 for reagents).

### 5.6 MDM and moDC Co‐Culture With Autologous T Cells

For co‐culture studies, moDCs or M2‐like MDM were generated as abovementioned. Differentiated cells were then co‐cultured with purified autologous T cells using the EasySep Human T cell isolation kit (STEMCELL Technologies) at a 1:2 macrophage to T cell ratio. T cells were stained with violet cell proliferation tracer (Invitrogen) prior to co‐culture. The co‐cultured cells were then stimulated either with CEFTA (Mabtech) or left unstimulated for 16 h or 5 days. For the 16 h cultures, Golgi‐Plug inhibitor Brefeldin A was added to cells 4 h before harvest, and cells were then stained for intracellular IL‐2 within CD4^+^ and CD8^+^ T cells, and supernatants were collected for detection of IL‐2 with ELISA. On day 5, cell culture supernatants were collected and stored at −20°C for the detection of cytokines using ELISA (Table 2). Harvested cells were stained for additional surface markers and analysed using flow cytometry (see co‐culture panel on Table 1 and Figure S6C).

### 5.7 Cytokine Detection by ELISA

Sandwich ELISA was performed for the detection of Cytokines (see Table 2) from the culture supernatant according to the manufacturer's instructions. Briefly, ELISA plates were coated overnight with the cytokine‐specific antibody, followed by the addition of culture supernatant and the standard to the plate. Biotinylated secondary antibody, followed by streptavidin conjugated ALP or HRP, was added to the plate. Substrate for the respective enzyme conjugate was added to the plate, and the reading was taken in the *V*_max_ microplate reader (Molecular Devices Corp.).

### 5.8 Statistical Analysis

GraphPad Prism 8 (GraphPad Prism Inc.) was used for the statistical analysis. Wilcoxon rank‐sum test or Kruskal–Wallis test followed by Dunn's multiple comparison was applied to determine the differences between the experimental groups. *p*‐values <0.05 were considered statistically significant. All plots show the median with interquartile range. Specific statistical tests can be found in the figure legends.

## Author Contributions

Claudia Arasa, Khaleda Rahman Qazi, Manuel Mata Forsberg, and Eva Sverremark‐Ekström conceptualized the study. Claudia Arasa and Khaleda Rahman Qazi performed all experiments and analyzed the data. David Brodin performed bioinformatics analysis and contributed to figure realization. Claudia Arasa and Eva Sverremark‐Ekström wrote the manuscript; all authors critically revised and accepted the manuscript.

## Conflicts of Interest

The authors declare no conflicts of interest.

## Ethics Statement

Peripheral blood mononuclear cells were obtained from healthy anonymous individuals following blood donations. No personal information can be traced back to the donor. Accordingly, the use of these residual blood products that would otherwise be discarded does not require ethical approval according to national regulations.

## Supporting information




**Supporting File 1**: eji70104‐sup‐0001‐SuppMat.pdf.

## Data Availability

The data that support the findings of this study are available from the corresponding author upon reasonable request.
