# Supplementary material for: Staphylococcal Enterotoxin A Shapes Monocyte Transcription and Macrophage Polarization: Implications for Immune Responses in Infection and Inflammation
Source: Eur J Immunol. 2025 Dec 19;55(12):e70104. doi: 10.1002/eji.70104 (PMC12716226; doi:10.1002/eji.70104)
Supplement: Supplementary file 1 — Supporting File 1: eji70104‐sup‐0001‐SuppMat.pdf. [file EJI-55-e70104-s001.pdf]

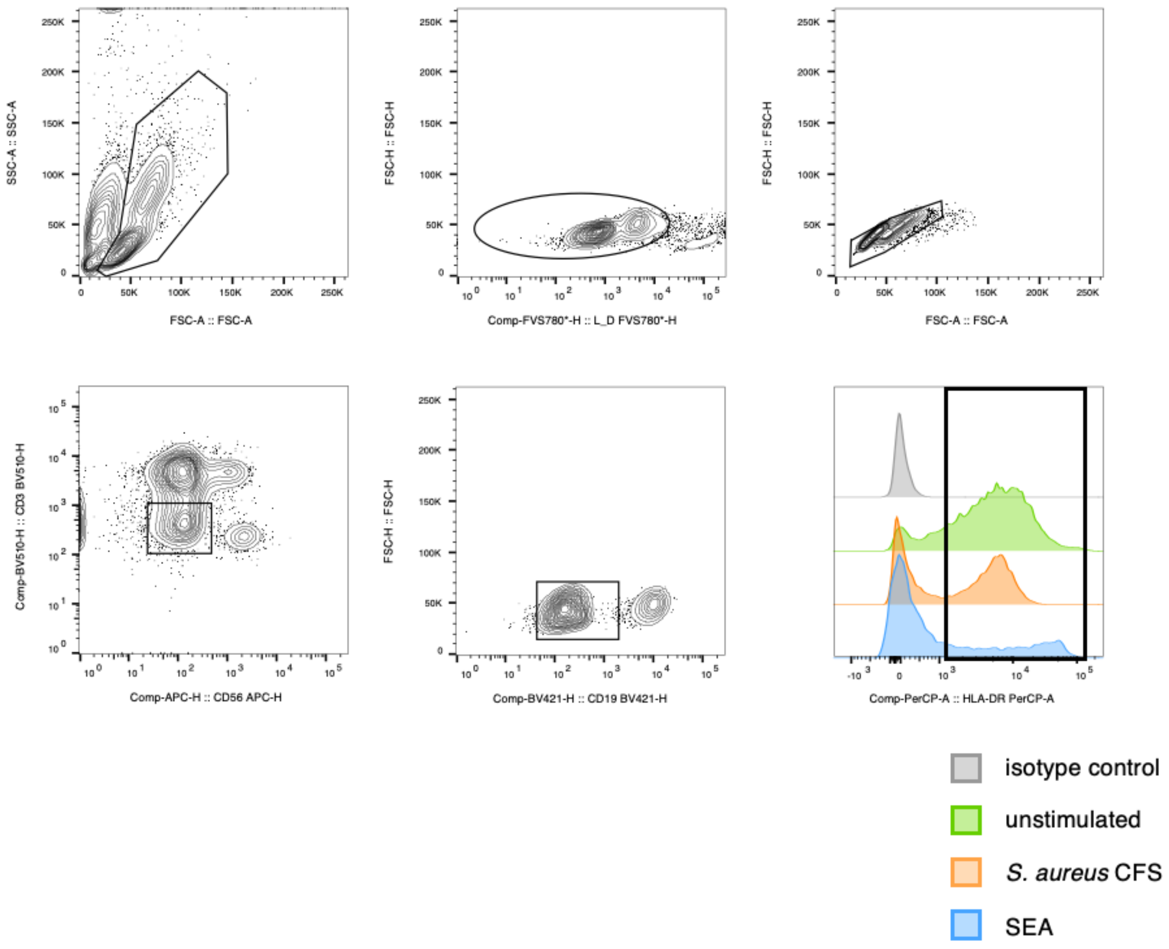

Supplementary Figure 1. Sequential gating strategy for the monocyte sorting after SEA stimulation of PBMC. Cells were first identified based on forward and side scatter properties, followed by the exclusion of dead cells using a viability dye. Single cells were then selected by gating on FSC-A versus FSC-H. Lymphocytes and NK cells were excluded by gating out $CD3^+$ and $CD56^+$ cells, and B cells were excluded by gating out $CD19^+$ cells. Finally, HLA-DR^+ cells were sorted.

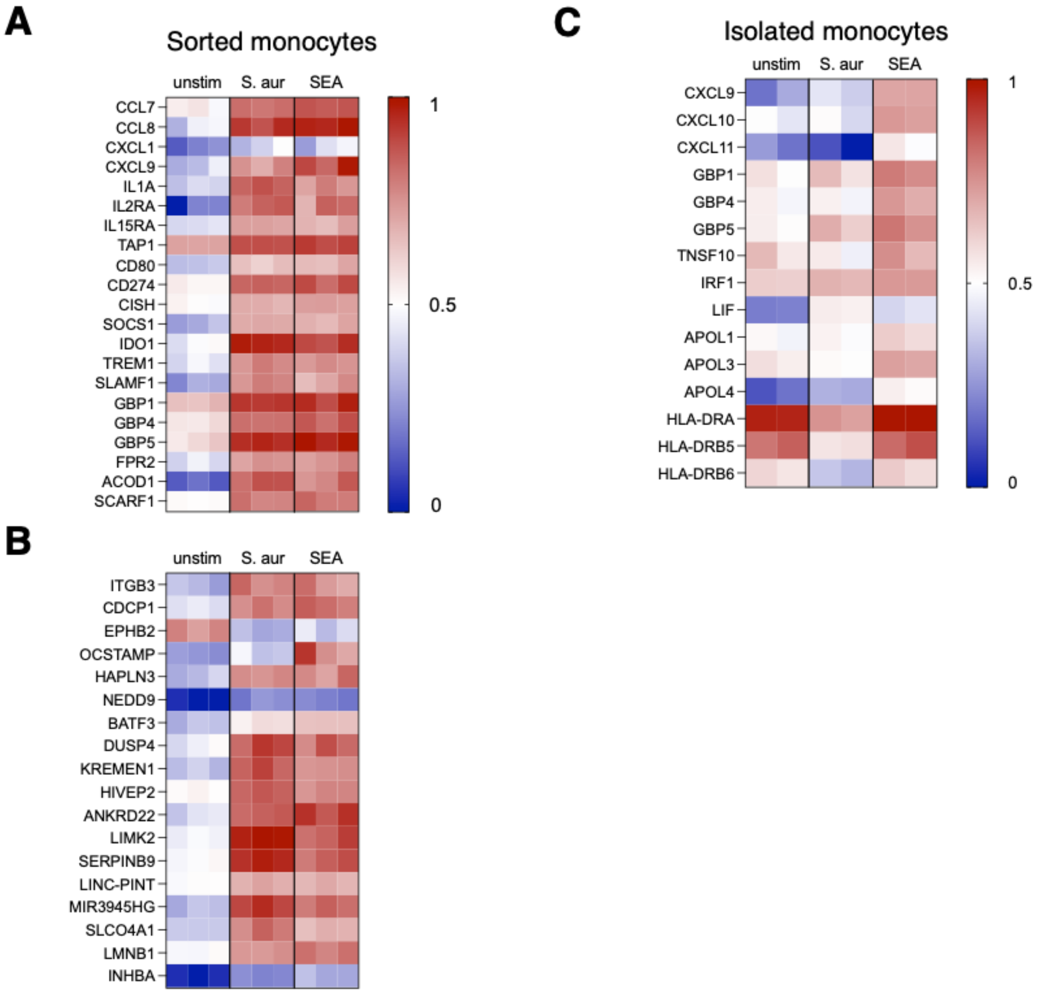

Supplementary Figure 2. Heatmaps of relative gene expression in sorted and isolated monocytes. Genes with a I log2FC>1 I and p adjusted <0.01 when comparing unstimulated with SEA-stimulated cells were selected. Normalized gene counts were first log2-transformed to reduce data skewness, and then normalized using Z-scores to place all genes on a unified scale to allow for direct comparison of expression patterns. (A) and (B) show the relative expression in sorted monocytes; (C) in isolated monocytes.

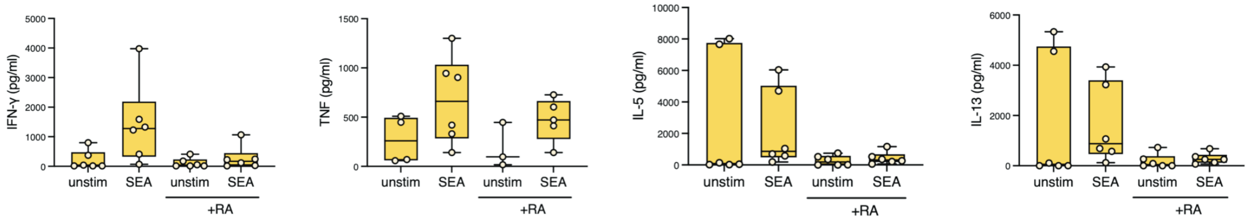

Supplementary Figure 3. T cell cytokine secretion upon moDC and T cell co-culture. Secretion levels of IFN-γ, TNF, IL-5 and IL-13 from co-culture of moDCs or gut-like moDCs with autologous T cells. The data are presented as median with interquartile range, with dots representing individual donors. Wilcoxon matched-pairs signed ranktest was used to determine statistical difference. P-values below 0.05 were considered statistically significant. Data were obtained from 6 donors and 3 independent experiments.

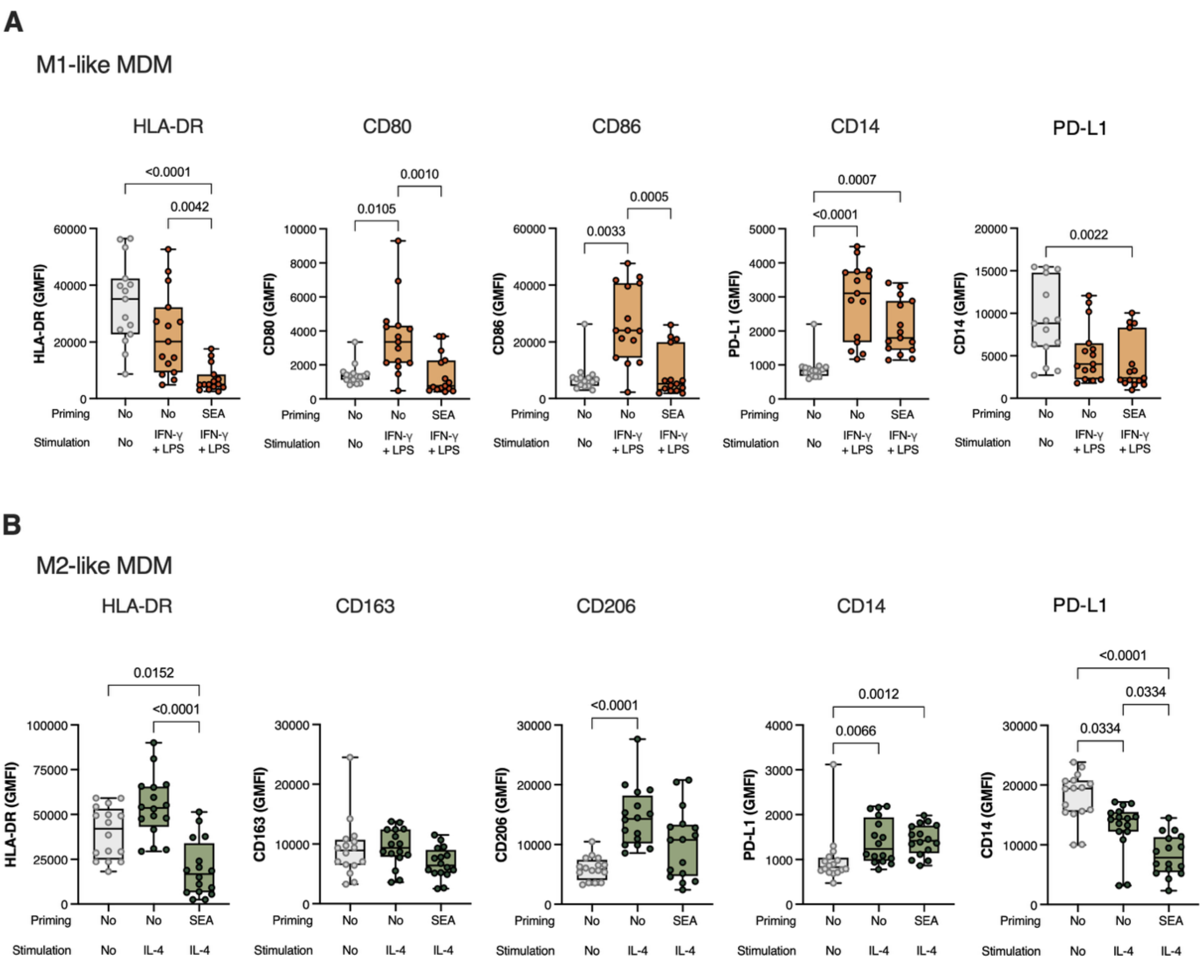

Supplementary Figure 4. Geometrical Mean Fluorescence Intensity (GMFI) of MDMs in M1- and M2-like conditions. (A) GMFI of HLA-DR, CD80, CD86, CD14 and PD-L1 in M1-like conditions; (B) GMFI of HLA-DR, CD163, CD206, CD14 and PD-L1 in M2-like conditions. The data are presented as median with interquartile range with dots representing individual donors. Kruskal-Wallis test was used to determine statistical differences, p-values below 0.05 were considered statistically significant and are written where appropriate. Data were obtained from 15-16 donors and 6 indepdendent experiments.

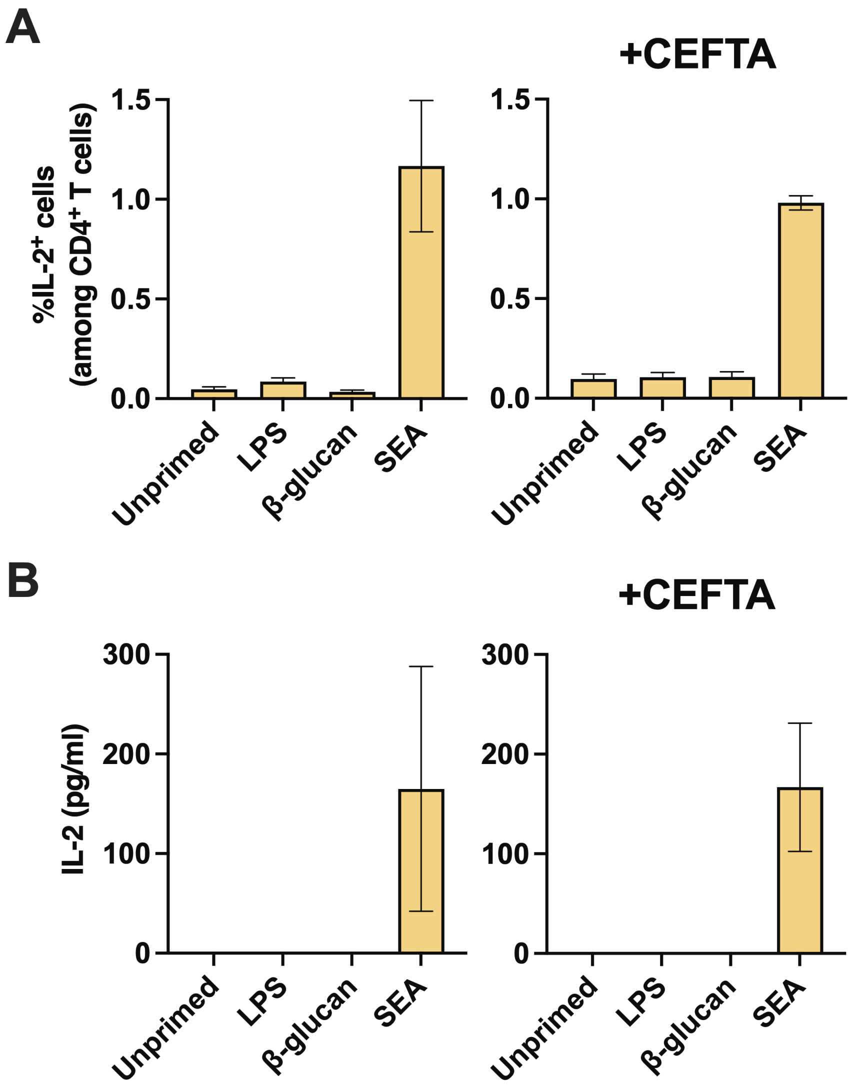

Supplementary Figure 5. IL-2 response in T cells co-cultured with MDM. Isolated monocytes were primed with LPS, β-glucan or SEA for 24 hours, washed and differentiated into M2-like monocyte-derived macrophages (MDM). Next, autologous CD3+ T cells were co-cultured with MDM in the presence or absence of 1 μg/mL CEFTA for 16 hours with the golgi-plug inhibitor Brefeldin A added during the last 4 hours of culture. A) Cells were collected and stained for intracellular IL-2 within CD4+ (Top) and CD8+ (Bottom) T cells and analysed with flow cytometry and B) secreted levels of IL-2 was quantified using ELISA. Figure represents one experiment with n=3 and data shows mean ± SEM.

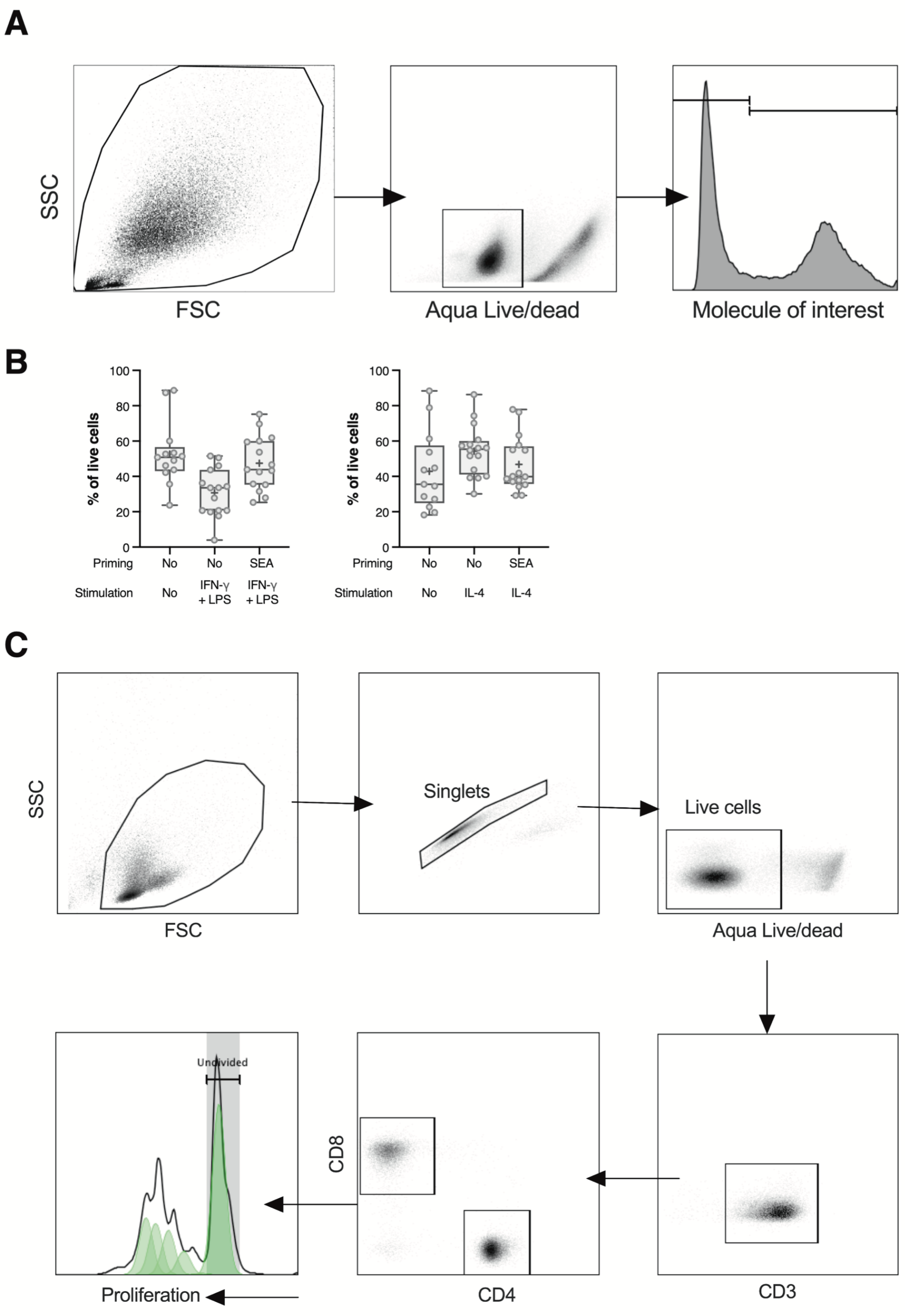

Supplementary Figure 6. Gating strategy for macrophage characterization and co-culture with autologous T cells. (A) Live, single MDMs were phenotyped by staining them with different surface markers conjugated with fluorochromes (see Table 1). Cells were first gated on the basis of their physical properties: forward scatter (FSC) and side scatter (SSC), followed by gating of live cells. Live cells were then analyzed for the expression of the molecules of interest as HLA-DR, CD14, CD80, CD86, CD163, CD206 and PDL-1. Geo MFI was calculated on the positive population. (B) Percentages of live cells in the M1- and M2-like conditions. (C) Schematic presentation of the gating strategies used for the identification of $CD4^+$ and $CD8^+$ T cells from live $CD3^+$ T cell gating in co-culture studies. FlowJo proliferation platform was used to calculate the frequencies of divided cells and division index.
